# Experiential Avoidance and Psychoactive Substance Use: Systematic Review

**DOI:** 10.3390/ejihpe16020022

**Published:** 2026-02-11

**Authors:** Gabriela Sequeda, Sandra Durán-Rondón, Johan E. Acosta-López, Eduardo-Andrés Torres-Santos, Diego Rivera-Porras

**Affiliations:** 1 Universidad Simón Bolívar, Facultad de Ciencias Jurídicas y Sociales, Grupo de Investigación en Modelamiento Científico e Innovación Empresarial, Cúcuta 540001, Colombia; sandra.duranr@unisimon.edu.co (S.D.-R.); eduardoa.torres@unisimon.edu.co (E.-A.T.-S.); 2 Universidad Simón Bolívar, Facultad de Ciencias Jurídicas y Sociales, Centro de Investigaciones en Ciencias de la Vida, Barranquilla 080005, Colombia; johan.acosta@unisimon.edu.co; 3 Universidad de la Costa, Departamento de Productividad e Innovación, Barranquilla 080001, Colombia

**Keywords:** experiential avoidance, substance use, addiction, emotion regulation, Acceptance and Commitment Therapy (ACT)

## Abstract

**Background**: Experiential avoidance (EA) refers to the tendency to evade or suppress unpleasant internal experiences, such as distressing thoughts, emotions, or bodily sensations. Increasing evidence indicates that EA plays a central role in the onset and maintenance of addictive behaviours. **Objective**: To synthesise quantitative evidence on the association between experiential avoidance (EA), operationalised as psychological inflexibility, and psychoactive substance use (PSU) outcomes, including substance use frequency/quantity, craving, dependence severity, relapse/abstinence, and treatment response, and to characterise putative pathways (EA as predictor/mediator) and correlates (e.g., affect regulation and trauma-related factors). **Methods**: A systematic search was conducted in SCOPUS, Web of Science, PubMed, and APA PsycNet, following PRISMA 2020 guidelines. Eligible studies included experimental and observational designs, clinical and non-clinical populations, and publications from January 2000 to January 2026 in English or Spanish. Primary outcomes were PSU behaviour and severity (frequency/quantity, craving, dependence symptoms, relapse/abstinence) and treatment outcomes; secondary outcomes included emotional and behavioural correlates linked to EA. **Results:** Across studies, higher levels of EA were consistently associated with greater substance use—particularly alcohol, tobacco, cannabis, and other illicit drugs. EA frequently mediated the relationships between emotional dysregulation, trauma exposure, and addictive behaviour. Elevated EA was also linked to impulsivity, psychiatric comorbidity, and poorer treatment adherence and outcomes. Interventions explicitly targeting EA—most notably Acceptance and Commitment Therapy (ACT)—showed promising effects in reducing avoidance and substance use. **Conclusions**: Experiential avoidance emerges as a transdiagnostic process underlying vulnerability to, and persistence of, substance use disorders. Integrating third-wave behavioural interventions that promote psychological flexibility may enhance the efficacy of addiction treatment. Future research should explore these mechanisms in culturally diverse and under-represented contexts.

## 1. Introduction

The United Nations Office on Drugs and Crime (UNODC, 2022) reports that more than 275 million people used drugs in 2020, with the highest prevalence in North America, South Asia, and Europe.

In 2019, the average per capita alcohol intake among adults (aged 15 years or older) was 5.5 litres of pure alcohol, equivalent to nearly two standard drinks per day for active consumers (World Health Organization, 2024). More than 52% of men and 35% of women reported alcohol use in the preceding year (World Health Organization, 2024). In the case of tobacco, approximately 1.3 billion people use tobacco products, 80% of whom reside in low- and middle-income countries where cessation resources remain scarce (World Health Organization, 2024). The World Health Organization attributes over seven million annual deaths to tobacco use, including 1.6 million resulting from second-hand smoke exposure (World Health Organization, 2024). Cannabis remains the most widely consumed illicit drug, with 147 million users annually, accounting for about 2.5% of the global population (World Health Organization, 2023). The health consequences of PSU extend beyond addiction itself, encompassing a spectrum of cardiovascular, respiratory, hepatic, metabolic, and neuropsychiatric pathologies that impose a heavy economic burden on healthcare systems and productivity worldwide.

### 1.1. Beyond Neurobiology: The Psychological Dimension of Addiction

Traditional models of addiction emphasised neurobiological mechanisms such as dopaminergic dysregulation, sensitisation of mesolimbic reward circuits, and maladaptive conditioning processes (Koob & Volkow, 2016). These frameworks elucidate the neurochemical underpinnings of craving, reinforcement, and withdrawal, yet often neglect the cognitive, emotional, and contextual factors that precipitate and perpetuate addictive behaviours. Over the past two decades, research in affective neuroscience and clinical psychology has underscored that addiction is not merely a pharmacological disorder of the brain’s reward system, but also a psychological disorder of emotion regulation (Garland et al., 2010; Paulus & Stewart, 2014). Individuals often engage in substance use as a maladaptive coping mechanism to modulate distress, escape aversive states, or suppress traumatic memories—processes conceptually aligned with the construct of experiential avoidance (EA).

### 1.2. Experiential Avoidance: Conceptualisation and Theoretical Framework

Experiential avoidance refers to a pervasive tendency to evade, suppress, or modify internal experiences, such as unwanted thoughts, emotions, memories, or bodily sensations, even when such strategies produce greater long-term suffering (Hayes et al., 1996; Hayes et al., 2006). EA represents a central dimension of psychological inflexibility, a construct fundamental to third-wave behavioural therapies, particularly Acceptance and Commitment Therapy (ACT). Although commonly described as “third-wave”, these interventions extend principles from both traditional behaviour analysis (first wave) and cognitive–behavioural therapy (second wave); their distinctive contribution lies in a contextual, process-based emphasis on psychological flexibility and on acceptance- and mindfulness-based methods (Hayes et al., 1996; Hayes et al., 2006). Within ACT, psychopathology is conceptualised as domination of avoidance and cognitive fusion over flexible, values-guided action. Through experiential avoidance, short-term relief from discomfort reinforces long-term maladaptive patterns, perpetuating cycles of dependence and distress.

Within the ACT literature, experiential avoidance (EA) is often operationalised as psychological inflexibility (PI), the converse of psychological flexibility (PF). These terms are closely related but not interchangeable: PF refers to the capacity to persist or change behaviour in the service of values while remaining open to internal experiences, whereas PI reflects rigid avoidance and behavioural inflexibility (Hayes et al., 2011). In this review, we treated EA/PI as conceptually aligned and standardised directionality so that higher scores consistently reflected greater avoidance/inflexibility (i.e., lower PF), regardless of whether a study reported PF or PI (Barrado-Moreno et al., 2025).

In the context of substance use, EA provides a compelling psychological account of why individuals persist in consumption despite awareness of harm. Substances serve as negative reinforcers, reducing emotional or physiological distress temporarily while strengthening avoidance-oriented coping styles (Levin et al., 2012). Over time, these patterns amplify impulsivity, diminish distress tolerance, and impair self-regulation, contributing to relapse vulnerability. Empirical studies demonstrate that individuals high in EA report greater cravings, lower treatment motivation, and higher relapse rates (Lappalainen et al., 2014; Shorey et al., 2017).

### 1.3. Emotional Dysregulation, Trauma, and Comorbidity

The relationship between EA and PSU is particularly salient among individuals with trauma histories or affective dysregulation. Exposure to childhood maltreatment, sexual abuse, or combat-related trauma has been consistently associated with elevated EA, and several studies suggest that EA can act as a mechanistic pathway linking trauma-related distress to substance misuse (Feingold & Zerach, 2021; Gratz et al., 2007; Klanecky et al., 2012). In specific populations (e.g., HIV-positive MSM using methamphetamine), trauma and experiential avoidance have also been examined in relation to disease management and substance use behaviours (Chartier et al., 2010). These individuals often experience heightened physiological arousal and intrusive memories, leading them to rely on substances as an avoidance mechanism. Furthermore, EA correlates with anxiety sensitivity, depressive symptomatology, and diminished emotion-regulation capacity, all of which exacerbate the progression of SUDs (Bakhshaie et al., 2016; Buckner & Zvolensky, 2014). Smoking-related inflexibility has also been proposed as a mechanism linking anxiety sensitivity to smoking severity (Jardin et al., 2015).

At the neurobiological level, avoidance-driven coping has been associated with alterations in prefrontal and limbic circuitry, particularly hypoactivation of regulatory regions such as the dorsolateral prefrontal cortex and hyperactivation of the amygdala during stress (Garland & Howard, 2018). This imbalance mirrors the behavioural pattern of avoiding emotional discomfort through immediate reinforcement—precisely the mechanism that underlies addictive cycles.

### 1.4. Clinical Implications and Evidence-Based Interventions

Addressing EA has become a central focus of third-wave behavioural interventions (Cavicchioli et al., 2020; Luoma et al., 2020; Twohig et al., 2007). Meta-analytic evidence indicates that ACT is associated with improvements in substance use outcomes (E. B. Lee et al., 2015), and mindfulness-based relapse prevention shows benefits in relapse-related outcomes (Grant et al., 2017). A recent systematic review and meta-analysis further supports the relevance of psychological flexibility/inflexibility in addiction phenotypes (Barrado-Moreno et al., 2025). Acceptance and Commitment Therapy (ACT) and Dialectical Behaviour Therapy (DBT) have demonstrated promising outcomes in reducing avoidance, enhancing emotional regulation, and improving abstinence rates across multiple substance types. ACT, for instance, facilitates acceptance of distressing internal experiences while promoting engagement with values-based goals, thereby weakening the reinforcement loop between avoidance and substance use. Similarly, DBT focuses on increasing tolerance for negative affect and developing mindfulness skills to replace impulsive coping with adaptive regulation (Linehan, 2015). These interventions highlight that reducing EA not only alleviates substance use but also enhances broader wellbeing and life functioning.

Nevertheless, the majority of empirical investigations into EA and PSU have been conducted in high-income Western contexts. The scarcity of research in low- and middle-income regions, including Latin America, constrains understanding of how sociocultural variables, such as collective coping, family cohesion, stigma, or socioeconomic adversity, modulate avoidance processes. This limitation underscores the need for systematic reviews that integrate cross-cultural evidence and identify common and context-specific pathways linking EA and substance use.

### 1.5. Rationale and Aims of the Present Review

Despite robust theory and a growing empirical literature, the evidence linking EA/psychological inflexibility to PSU remains dispersed across substances, populations, and study designs, and existing reviews have typically focused on specific interventions (e.g., ACT/MBRP) rather than EA as a process-based mechanism across substances.

Accordingly, this systematic review aims to consolidate quantitative evidence on EA/psychological inflexibility in relation to PSU outcomes (use level and severity, craving, relapse/abstinence, and treatment response) across alcohol, tobacco/nicotine, cannabis, cocaine, opioids, and polysubstance use.

Specifically, the review synthesises (i) the direction and magnitude of associations between EA and PSU outcomes, (ii) evidence for EA as a predictor or mediator of PSU-related risk and maintenance, and (iii) the psychological and clinical correlates most consistently co-occurring with EA in PSU contexts.

Research question: In clinical and non-clinical populations using psychoactive substances, how is EA/psychological inflexibility associated with PSU outcomes (use frequency/quantity, craving, dependence severity, relapse/abstinence) and related psychological correlates?

## 2. Materials and Methods

This systematic review was conducted in strict adherence to the Preferred Reporting Items for Systematic Reviews and Meta-Analyses (PRISMA 2020) guidelines (Page et al., 2021), which provide a methodological framework that guarantees transparency, reproducibility, and rigour throughout all stages of the review process. The purpose of this study was to identify, analyse, and synthesise empirical evidence examining the relationship between experiential avoidance (EA) and psychoactive substance use (PSU) in both clinical and non-clinical populations.

The methodological design followed the PRISMA structure, including the stages of identification, screening, eligibility, and inclusion. Each article was reviewed in detail to extract information related to authorship, publication year, target population, study design, instruments, variables, and main findings.

### 2.1. Design and Scope of the Review

A systematic and analytical design was employed to capture quantitative empirical studies examining the relationship between experiential avoidance (EA) and psychoactive substance use (PSU). The search covered publications from January 2000 to January 2026 across SCOPUS, Web of Science, PubMed, and APA PsycNet, complemented by targeted update search and citation tracking (January 2026) to identify recent or overlooked studies. Psychoactive substances of interest included alcohol, tobacco/nicotine, cannabis, cocaine, opioids, and polysubstance use, consistent with the categorisation used in the data extraction tables.

Two independent reviewers conducted screening and data extraction in duplicate. Discrepancies in inclusion or exclusion decisions were discussed and resolved by consensus with a third reviewer. The scope included studies that evaluated EA either as a risk factor, mediator, or maintaining variable in the use of alcohol, tobacco, cannabis, opioids, or other psychoactive substances. Both observational and interventional studies were eligible provided that they offered original quantitative data and a clear conceptualisation of EA.

**Protocol Registration**

This systematic review was prospectively registered in the International Prospective Register of Systematic Reviews (PROSPERO) to ensure methodological transparency and to minimise the risk of duplication within the scientific community. The protocol was registered under the title “Experiential Avoidance and Psychoactive Substance Use: Systematic Review”, with the registration number CRD420251117875. The registration includes the definition of the research question, eligibility criteria, search strategy, study selection procedures, data extraction plan, and synthesis methods established prior to commencing the review, in accordance with PRISMA 2020 guidelines.

### 2.2. Formulation of the Research Question

The guiding research question was structured using the PICOS framework (Population, Intervention/Exposure, Comparator, Outcomes, and Study design), which is widely used to guide systematic reviews and to pre-specify eligibility and extraction in a replicable manner. Because our question concerns an exposure/process rather than a randomised intervention, the “I” component is operationalised as exposure to higher EA/psychological inflexibility.

Population (P): clinical and non-clinical groups (adolescents and adults) reporting PSU; Intervention/Exposure (I/E): EA/psychological inflexibility assessed with validated instruments (e.g., AAQ-II, MEAQ, AAQ-SA) or clearly defined operationalisations; Comparator (C): lower EA/greater psychological flexibility, or between-group contrasts defined within studies; Outcomes (O): PSU behaviour and severity (frequency/quantity, craving, dependence symptoms, relapse/abstinence) and treatment outcomes; Study design (S): quantitative observational or experimental studies.

The research question was thus formulated as follows: In clinical and non-clinical populations using psychoactive substances, how is EA/psychological inflexibility (vs. lower EA/greater flexibility) associated with PSU outcomes and related psychological correlates?. The PICOS structure of the research question is presented in Table 1.

### 2.3. Search Strategy and Information Sources

A comprehensive and systematic search was conducted across four electronic databases—PubMed, SCOPUS, Web of Science (WoS), and APA PsycNet—chosen for their extensive coverage of psychological, clinical, and behavioural sciences.

Search algorithms were developed using terms from the Medical Subject Headings (MeSH) and Descriptores en Ciencias de la Salud (DeCS) thesauri. Boolean operators (“AND”, “OR”) were used to combine descriptors, and quotation marks were employed to ensure precision in phrase matching. Only studies published in English or Spanish were included, and filters were applied to restrict the results to empirical, peer-reviewed journal articles. Reviews, theoretical essays, editorials, conference abstracts, and documents without full-text access were excluded. An updated search was conducted in January 2026, adding ‘psychological flexibility’ as a search term and using citation tracking to identify additional eligible studies.

The main conceptual categories and their corresponding descriptors are presented below. The DeCS and MeSH descriptors used in the search strategy are presented in Table 2.

For transparency and replicability, the database-specific Boolean strings are reported in full in Table 3.

### 2.4. Eligibility Criteria

Studies were selected according to pre-specified inclusion and exclusion criteria defined prior to screening.

Inclusion criteria were as follows:

(a) Experiential avoidance or psychological inflexibility was included as a measured or theoretically central construct.
(b) The population included participants with psychoactive substance use, either in clinical or community settings.
(c) The study presented original quantitative empirical data (cross-sectional, longitudinal, experimental, or quasi-experimental).
(d) The study reported at least one quantitative PSU outcome (e.g., use frequency/quantity, craving, dependence severity/symptoms, relapse/abstinence) or a treatment outcome relevant to PSU (e.g., adherence, response, abstinence).
(e) The article was published between 2000 and January 2026 in a peer-reviewed journal, in English or Spanish.
(f) The publication contained sufficient methodological information for critical appraisal.
At full-text screening, reports were excluded if they met any exclusion criterion (e.g., reviews/meta-analyses, qualitative-only studies without quantitative PSU outcomes, behavioural addictions only, no EA/psychological (in)flexibility measure or operational definition, outcomes unrelated to PSU, inaccessible/incomplete full texts, or duplicate datasets).

Exclusion criteria were as follows:

(a) Review articles, meta-analyses, editorials, protocols, or purely theoretical papers; (b) qualitative-only studies without quantitative PSU outcomes; (c) studies focusing exclusively on behavioural addictions (e.g., gambling, internet use) without psychoactive substances; (d) studies that did not measure EA/psychological (in)flexibility or did not provide an operational definition; (e) studies unrelated to PSU outcomes; and (f) inaccessible or incomplete full texts preventing extraction.

### 2.5. Study Identification and Screening Process

The initial search retrieved 431 records across the four databases (Scopus = 78, PubMed = 102, Web of Science = 217, and APA PsycNet = 34). To address potential omissions in newer literature and terminological variability (e.g., ‘psychological flexibility’), we performed citation tracking and an updated targeted search in January 2026, which identified one additional eligible record (*n* = 1). All records were exported to Rayyan QCRI software version 1.7.2 (as of February 2026) to facilitate screening, deduplication, and tracking. After removing 159 duplicates, 273 unique records remained. Title and abstract screening excluded 112 records, leaving 161 reports for full-text assessment. Following full-text eligibility assessment, 120 reports were excluded, and 41 studies were retained for final synthesis. 

The quantitative summary of the selection process is shown below. The database-specific contribution to records identified, deduplication, screening, and included studies is presented in Table 4.

**Figure 1:**
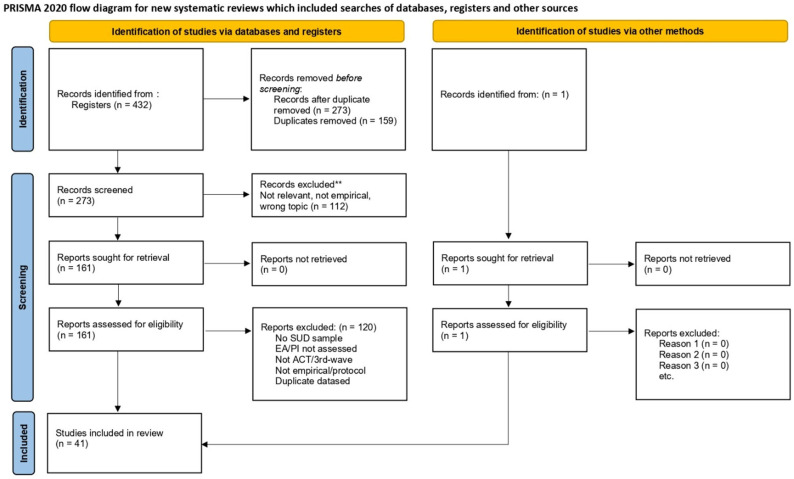
PRISMA 2020 flowchart, showing the process of search, selection, inclusion and exclusion of articles. Source: PRISMA 2020. Updated to reflect revised counts after targeted update search and citation tracking (January 2026). ** Records excluded after title/abstract screening due to irrelevance to the research question, non-empirical design, or unrelated topic.

The distribution revealed that Web of Science produced the highest number of initial records but also the highest redundancy rate. PubMed and Scopus contributed substantially to the final sample, while APA PsycNet, despite a smaller corpus, yielded thematically concentrated and methodologically strong studies. The PRISMA 2020 checklist is provided as Supplementary Material (See Supplementary Materials). The selection flow adhered to PRISMA standards and is illustrated in Figure 1.

### 2.6. Data Extraction and Quality Appraisal

Data were extracted using a structured Excel matrix that recorded the bibliographic metadata, study design, participant characteristics, type of substance, instruments measuring EA, psychological correlates, and main results. Two reviewers independently performed the extraction and cross-verified entries to ensure accuracy.

Quality assessment followed a modified version of the Joanna Briggs Institute (JBI) critical appraisal checklist, evaluating the clarity of aims, adequacy of sample size, validity of instruments, control of confounders, transparency of statistical analyses, data completeness, and acknowledgment of limitations. Studies meeting at least five criteria were classified as high quality, those meeting three or four as moderate, and fewer than three as low. Approximately 75% of the studies demonstrated moderate to high methodological quality.

### 2.7. Ethical Considerations

This review involved secondary data only; therefore, no direct participation of human subjects occurred, and ethical approval was not required. However, all included studies reported institutional ethics approval and compliance with the Declaration of Helsinki.

### 2.8. Data Synthesis

A narrative synthesis approach was adopted due to heterogeneity across study designs, measures, and outcomes. Studies were grouped by substance type (alcohol, tobacco, cannabis, opioids, and polysubstance use) and by psychological mechanisms (emotional dysregulation, impulsivity, trauma, and treatment response). This structure facilitated a transdiagnostic interpretation of experiential avoidance, highlighting its mediating and maintaining role in substance use behaviours.

The results of this synthesis, organised by substance type and theoretical domain, are detailed in the following section (Results), providing an integrative understanding of how experiential avoidance interacts with affect regulation and substance-related patterns.

Methodological characteristics: The search was updated through January 2026; no additional eligible studies beyond 2024 were identified.

The systematic review included a total of 41 studies published between 2003 and 2024, with various methodological approaches (Forsyth et al., 2003; Levin et al., 2012; Serowik & Orsillo, 2019; Mak et al., 2021; Hooper et al., 2018; Farris et al., 2016a, 2016b; Stotts et al., 2015; Vernig & Orsillo, 2009; Klanecky et al., 2012). Cross-sectional and correlational designs predominated, although controlled clinical trials and experimental studies were also identified. Pre–post assessments and secondary analyses of data from clinical trials were also reported. Sample sizes varied widely, from small student samples (*n* = 48; *n* = 54) to samples of several hundred participants (*n* = 465; *n* = 298).

These studies included both clinical populations undergoing treatment for addiction (Brem et al., 2017b; Elmquist et al., 2018; Naifeh et al., 2012) and samples of young adult university students with varying levels of consumption (Karekla et al., 2017; Klanecky et al., 2012; Levin et al., 2012; Serowik & Orsillo, 2019). In general, most studies measured experiential avoidance using standardised inflexibility or avoidance questionnaires (typically the Acceptance and Action Questionnaire-II or other derived scales) along with instruments to assess psychological symptoms and patterns of substance use. Few studies included psychophysiological measures; one exception was Vernig and Orsillo (2009), who assessed autonomic emotional responses. Most of the research used self-report data collected at a single point in time or in brief pre–post assessments. Only a few studies incorporated interventions aimed at modifying experiential avoidance or explicitly assessed their therapeutic effects on consumption, for example Hooper et al. (2018), Mak et al. (2021), and Stotts et al. (2015), while the rest examined associations between avoidance and other variables in natural samples.

Table 5 presents the included studies grouped by the main psychoactive substance (alcohol, tobacco/nicotine, cannabis, cocaine, opioids, or polysubstance use). Each study is summarised in one row reporting authors (year), country, design, sample, measures/procedures, key variables, and conclusions.

## 3. Results

### 3.1. Overview of Included Studies: The Search Was Updated Through January 2026; No Additional Eligible Studies Beyond 2024 Were Identified

The final sample comprised 41 empirical studies published between 2003 and 2024 (Buckner et al., 2014; Cavicchioli et al., 2020; Fazeli Rad et al., 2024; Hall et al., 2018; Luoma et al., 2020; Martínez-Vispo et al., 2020). Most were conducted in the United States (28/41, 68.3%), followed by Australia (3/41, 7.3%) and the United Kingdom (2/41, 4.9%); one study each was conducted in Israel, South Korea, Italy, Spain, Hong Kong, Cyprus, Iran, and Türkiye (each 1/41, 2.4%). Regarding study design, 26/41 (63.4%) were cross-sectional/correlational or secondary analyses, 9/41 (22.0%) were intervention, pilot, or pre–post studies, 4/41 (9.8%) were experimental or laboratory studies, and 2/41 (4.9%) were prospective or diary-based designs.

Participant samples were diverse and included university students, patients in outpatient or residential treatment programmes, smokers attempting cessation, combat veterans, individuals with psychiatric comorbidities such as borderline personality disorder or schizophrenia, and community participants (Farris et al., 2016a; Feingold & Zerach, 2021; Kingston et al., 2010).

The most frequently used measures of experiential avoidance (EA) were the Acceptance and Action Questionnaire-II (AAQ-II) and the Multidimensional Experiential Avoidance Questionnaire (MEAQ), while emotion regulation was typically measured using the Difficulties in Emotion Regulation Scale (DERS). Substance use was assessed through the AUDIT (alcohol), FTND (tobacco), and DUDIT (other drugs), together with affective and anxiety scales such as PANAS, BDI-II, CES-D, and ASI-3. Specific measures such as the Avoidance and Inflexibility Scale (AIS) for smoking and the Drinking Motives Questionnaire (DMQ) were also employed.

Most studies applied mediation models or structural equation modelling (SEM) to examine the relationships among trauma exposure, experiential avoidance, emotion dysregulation, and problematic substance use (Dvorak et al., 2013; E. S. Lee & Bong, 2018). In studies addressing stimulant or opioid use, distress tolerance and anxiety sensitivity emerged as significant mechanisms influenced by EA (Fazeli Rad et al., 2024; Naifeh et al., 2012).

Third-wave behavioural interventions, including Acceptance and Commitment Therapy (ACT) and Dialectical Behaviour Therapy (DBT), consistently demonstrated reductions in experiential avoidance and emotional dysregulation, although results for short-term abstinence were mixed (Cavicchioli et al., 2020; Hall et al., 2018; Mak et al., 2021; Fowler et al., 2016; Buckner et al., 2014; Dvorak et al., 2013; Gratz et al., 2007). In residential clinical contexts, reductions in EA were associated with improved emotional regulation and lower relapse rates. Collectively, the findings identify EA as a transdiagnostic process that contributes to the initiation, maintenance, and treatment response of psychoactive substance use, moderated by contextual, clinical, and individual factors such as severity, comorbidity, anxiety sensitivity, impulsivity, personality traits, and trauma history. Table 6 summarises the general characteristics of included studies.

### 3.2. Findings by Substance Type

#### 3.2.1. Alcohol

The ten studies focused on alcohol (IDs 1–10) consistently indicated that higher experiential avoidance was associated with greater alcohol-use severity, coping-motivated drinking, and negative affect.

Among young adults and university students, higher levels of EA and negative affect predicted a greater likelihood of solitary drinking and higher alcohol intake (Levin et al., 2012; Martínez-Vispo et al., 2020).

Mediation analyses showed that childhood trauma predicted alcohol-related problems through experiential avoidance and emotional dysregulation, with differential patterns according to post-traumatic stress severity (Dvorak et al., 2013; Klanecky et al., 2012).

In combat veterans, EA was positively associated with PTSD symptoms, emotional suppression, and lower levels of cognitive reappraisal (Feingold & Zerach, 2021).

Experimental studies with alcohol-dependent participants demonstrated stronger avoidance and escape tendencies (Sheynin et al., 2019), whereas DBT programmes led to significant reductions in both EA and emotional dysregulation, thereby supporting abstinence (Cavicchioli et al., 2020).

Overall, the evidence identifies experiential avoidance as a maintaining mechanism linking trauma, anxiety, and impulsivity to maladaptive alcohol use. Moreover, interventions emphasising acceptance, mindfulness, and emotion regulation have shown clinical efficacy in decreasing consumption frequency and enhancing self-regulation (Luoma et al., 2020; Vernig & Orsillo, 2009. Table 7 reports alcohol-related variables, methods, and key findings (IDs 1–10).

#### 3.2.2. Tobacco

The eleven studies addressing tobacco use (IDs 11–21) collectively indicated that experiential avoidance (EA) plays a significant role in the initiation, maintenance, and relapse of smoking behaviour (Farris et al., 2016b; Robles et al., 2016; Watson et al., 2017). Across clinical and non-clinical samples, higher EA was consistently linked to greater nicotine dependence, reduced abstinence rates, stronger craving, and greater difficulty managing negative affect.

Cross-sectional and correlational studies demonstrated that smokers with higher levels of avoidance reported greater perceived barriers to cessation, poorer distress tolerance, and stronger smoking motives related to anxiety or dysphoria (Farris et al., 2016a; Garey et al., 2016).

Among trauma-exposed or high-anxiety populations, anxiety sensitivity predicted smoking severity through EA, suggesting that avoidance mediates the emotional amplification of craving (Bakhshaie et al., 2016).

Furthermore, individuals with high social anxiety exhibited higher levels of smoking-specific EA and were more likely to engage in smoking as a means of avoiding aversive internal states, reinforcing dependence (Watson et al., 2017). In related work, social avoidance has been linked to substance use variability within social anxiety presentations (Aurora & Coifman, 2021).

Experimental and intervention studies further supported these associations.

Hooper et al. (2018) found that a cognitive defusion exercise, which targets avoidance tendencies, increased motivation to quit compared with control or pure avoidance conditions (Hooper et al., 2018).

Farris et al. (2016a) reported that sustained abstinence was accompanied by reductions in smoking-specific EA, particularly among women, who also presented higher baseline avoidance levels (Farris et al., 2016a).

Randomised and feasibility trials confirmed the efficacy of Acceptance and Commitment Therapy (ACT) in reducing EA and enhancing psychological flexibility, even though some trials did not yield significant changes in quit rates, likely due to small sample sizes or brief interventions (Mak et al., 2021).

At a clinical level, emotion dysregulation and avoidance coping were consistently related to relapse risk, depressive symptoms, and diminished environmental reward (Martínez-Vispo et al., 2020).

Physiological studies using laboratory paradigms (e.g., stress-induction via PASAT-C) revealed that stress exposure increased craving intensity and negative affect, reinforcing the function of smoking as an avoidance-based coping mechanism (Karekla et al., 2017; Karekla & Panayiotou, 2011).

Taken together, the evidence identifies experiential avoidance as a transdiagnostic process that sustains nicotine dependence through affective and cognitive mechanisms. Interventions that enhance psychological flexibility and reduce avoidance (such as ACT, defusion training, or emotion-regulation skills programmes) appear to offer clinically meaningful benefits, particularly for individuals with high anxiety sensitivity or emotional vulnerability. Table 8 reports tobacco/nicotine-related variables, methods, and key findings (IDs 11–21).

#### 3.2.3. Cannabis

The two studies focusing on cannabis use (IDs 22–23) consistently demonstrated that experiential avoidance (EA) is closely linked to the maintenance of cannabis dependence, particularly when use serves to regulate social anxiety and negative affect (Buckner & Zvolensky, 2014; Twohig et al., 2007). Although the sample size across these studies was limited, the convergent evidence highlights the avoidance of internal distress as a psychological mechanism underlying both the initiation and persistence of cannabis consumption.

Buckner and Zvolensky (2014) examined 123 regular cannabis users and found that individuals who reported high social anxiety were more likely to use cannabis as a strategy to avoid negative internal experiences, such as fear of social evaluation or physiological arousal. This avoidance-based motivation was significantly associated with greater dependence severity and lower self-efficacy for control. The authors concluded that the reinforcing function of cannabis use in these individuals lies primarily in its negative-reinforcement value, reducing aversive emotional states rather than enhancing pleasure (Buckner & Zvolensky, 2014).

Complementarily, Twohig et al. (2007) conducted a multiple-case pilot study evaluating the efficacy of Acceptance and Commitment Therapy (ACT) in three adults with cannabis dependence. Over eight ACT sessions, participants demonstrated notable reductions in experiential avoidance and increases in psychological flexibility, which corresponded with abstinence maintenance in two of the three cases and only one brief relapse. This preliminary evidence supports the hypothesis that interventions targeting avoidance processes can effectively disrupt the reinforcing cycle of cannabis use (Twohig et al., 2007).

Overall, these findings indicate that cannabis use may function as an avoidance-oriented coping strategy, particularly among individuals with social anxiety or heightened emotional reactivity. ACT-based approaches appear promising in addressing these patterns by fostering acceptance and non-judgemental awareness of internal experiences, thereby reducing reliance on substances for emotional regulation. Table 9 reports cannabis-related variables, methods, and key findings (IDs 22–23).

#### 3.2.4. Cocaine

The two studies examining cocaine use (IDs 24–25) revealed a robust association between experiential avoidance (EA), emotional distress, and treatment outcomes. Both studies converge on the notion that avoidance-oriented coping strategies hinder recovery and contribute to the persistence of dependence and relapse vulnerability (Naifeh et al., 2012; Stotts et al., 2015).

Stotts et al. (2015) conducted a post hoc analysis of a contingency management (CM) intervention involving 99 outpatient participants with cocaine dependence. The study demonstrated that individuals with high baseline levels of EA exhibited poorer treatment outcomes, including lower abstinence rates and reduced responsiveness to CM incentives. These findings suggest that avoidance may undermine behavioural reinforcement contingencies by limiting engagement with treatment demands and exposure to discomfort associated with abstinence. EA thus functions as a barrier to adaptive coping, reinforcing maladaptive emotional regulation patterns that sustain substance use (Stotts et al., 2015).

Similarly, Naifeh et al. (2012) examined 62 inpatients with comorbid trauma exposure and cocaine dependence. Results showed that emotional avoidance and anxiety sensitivity were significantly correlated with post-traumatic stress symptom severity and substance use intensity. The findings underscore the bidirectional relationship between trauma-related avoidance and cocaine dependence, whereby avoidance maintains both PTSD symptomatology and substance-related reinforcement. The authors argue that interventions targeting emotional acceptance and distress tolerance may help disrupt this cycle (Naifeh et al., 2012; Stotts et al., 2015).

The evidence highlights that experiential avoidance acts as a maladaptive mediator between trauma, anxiety sensitivity, and cocaine dependence. Therapeutic approaches incorporating acceptance, exposure, and mindfulness—such as Acceptance and Commitment Therapy (ACT) or exposure-based CM protocols—are recommended to enhance engagement and reduce relapse risk in this population. Table 10 reports cocaine-related variables, methods, and key findings (IDs 24–25).

#### 3.2.5. Opioids

The single study addressing opioid use (ID 40) provided valuable empirical evidence on the interplay between experiential avoidance (EA), distress tolerance, and craving among individuals undergoing long-term treatment for opioid use disorder (Fazeli Rad et al., 2024). Although limited in number, the findings add to the growing literature recognising avoidance processes as a central mechanism sustaining opioid dependence and emotional dysregulation.

Conducted in Iran with 241 adult men enrolled in residential treatment programmes for at least eight years, Fazeli Rad et al. (2024) employed a cross-sectional design to assess the mediating role of EA in the relationship between distress tolerance and craving intensity. Using validated instruments—including the Acceptance and Action Questionnaire-II (AAQ-II), Distress Tolerance Scale (DTS), and Desire for Drug Questionnaire (DDQ)—the authors observed that individuals with low distress tolerance exhibited higher experiential avoidance, which in turn predicted stronger drug craving and diminished emotional control (Fazeli Rad et al., 2024).

The mediation model demonstrated that EA acted as a psychological bridge between emotional vulnerability and craving maintenance. This suggests that individuals who attempt to suppress or escape aversive internal experiences are more likely to experience persistent craving cycles, sustaining the addictive behaviour. Moreover, emotional dysregulation, assessed via the Difficulties in Emotion Regulation Scale (DERS), amplified this association, underscoring the transdiagnostic nature of avoidance processes across emotional and behavioural domains.

These results underscore the relevance of acceptance-based interventions—particularly Acceptance and Commitment Therapy (ACT) and Dialectical Behaviour Therapy (DBT)—as complementary strategies to enhance distress tolerance, reduce experiential avoidance, and promote psychological flexibility among individuals recovering from opioid use. By improving tolerance for negative affect and disrupting maladaptive avoidance patterns, such interventions can contribute to craving reduction and long-term abstinence maintenance. Table 11 reports opioid-related variables, methods, and key findings (ID 40).

#### 3.2.6. Polysubstance and Multiple Substance Use

The largest thematic cluster within this review comprises fifteen studies (IDs 26–39) that investigated experiential avoidance (EA) in relation to multiple or polysubstance use disorders (SUDs). These studies collectively provide strong empirical support for the notion that EA operates as a transdiagnostic process that contributes to the onset, maintenance, and relapse across diverse substance classes. They also highlight the role of emotional dysregulation, trauma, impulsivity, and maladaptive coping as key mechanisms linking avoidance to addictive behaviour.

Across both clinical and non-clinical populations, EA consistently correlated with greater emotional distress, psychopathology, and impaired self-regulation. For instance, Serowik and Orsillo (2019) demonstrated among 223 students that individuals with higher EA scores and stronger coping-motivated substance use patterns reported greater alcohol and drug-related problems. Conversely, value-driven behaviour—indicative of lower experiential avoidance—was inversely related to substance misuse, suggesting that commitment to personally meaningful goals serves as a protective factor (Serowik & Orsillo, 2019).

In clinical samples, several studies confirmed EA’s role in co-occurring psychiatric symptoms. Brem et al. (2017a, 2018) and Elmquist et al. (2018) found that avoidance mediated the relationship between shame, trauma, and compulsive sexual or bulimic behaviours among men and women in residential SUD treatment. Brem et al. (2018) identified shame and PTSD as major predictors of avoidance and compulsive sexual behaviour, underscoring the need for trauma-informed approaches in addiction treatment. Similarly, Hall et al. (2018) reported that ACT-based interventions significantly reduced emotional dysregulation and avoidance in patients with comorbid borderline personality disorder (BPD) and SUDs, although high dropout rates limited generalisability.

In veterans and forensic populations, studies reported that EA was strongly associated with low perceived control, depression, and thought suppression, mechanisms that reinforce both emotional and behavioural rigidity (Chapman & Cellucci, 2007; Forsyth et al., 2003). Kingston et al. (2010) further suggested that avoidance may mediate the relationship between childhood trauma, affect intensity, and risky behaviour, reflecting the pervasive influence of avoidance as a maladaptive regulation strategy.

Finally, Gratz et al. (2007) and Baker et al. (2007) provided complementary insights into the emotional underpinnings of EA. Gratz et al. found that childhood abuse severity predicted higher avoidance and emotional non-acceptance, while Baker et al. observed that participation in music therapy sessions led to immediate improvements in mood and emotional expression, suggesting that experiential engagement may counteract avoidance-driven withdrawal patterns.

Together, these findings reveal that EA not only predicts substance use severity but also interacts with co-occurring affective disturbances to perpetuate addiction cycles. Interventions promoting acceptance, mindfulness, and value-oriented behaviour—such as Acceptance and Commitment Therapy (ACT)—demonstrate considerable promise in reducing avoidance, improving emotion regulation, and enhancing treatment adherence across multiple substance use contexts. Table 12 reports polysubstance and multiple substance use variables, methods, and key findings (IDs 26–39).

## 4. Discussion

The synthesis of 41 empirical studies published between 2003 and 2024 suggests consistent evidence that experiential avoidance (EA) is associated with the initiation, maintenance, and relapse-related processes of psychoactive substance use (PSU). Across different substances and populations, EA appears to function as a transdiagnostic vulnerability factor that may interact with emotion dysregulation, trauma exposure, and coping motives, potentially reinforcing addictive behaviours and reducing engagement with treatment.

### 4.1. Cross-Substance Patterns and Theoretical Convergence

Despite methodological variability among the studies—ranging from cross-sectional surveys to randomised clinical trials—the general pattern is remarkably consistent: higher levels of EA are positively associated with substance use frequency, dependence severity, and relapse vulnerability.

Across alcohol, tobacco, cannabis/cocaine, opioids, and polysubstance samples, higher EA was generally linked to more frequent use, greater severity, stronger craving, and higher relapse vulnerability; these associations were often embedded within broader affect-regulation processes (e.g., coping motives, anxiety, distress tolerance, and trauma-related symptoms). Given that the evidence base is heterogeneous and often cross-sectional, these patterns should be interpreted as associations rather than causal effects; study-level details are reported in the Results section (Table 6 and Table 11).

Collectively, these patterns are consistent with models of psychological inflexibility and emotion regulation, in which addiction can be conceptualised as a behavioural manifestation of experiential control—the chronic attempt to suppress or escape aversive private experiences (Hayes et al., 2011; Ehman & Gross, 2019). Empirically, psychological inflexibility has been linked to relapse risk and poorer treatment outcomes (Ulusoy et al., 2022).

### 4.2. Emotional Dysregulation and Trauma as Co-Factors

A cross-substance analysis indicates that EA seldom operates in isolation; rather, it co-occurs with emotional dysregulation and trauma history, forming an interactive triad that predicts both the onset and chronicity of PSU. Individuals exposed to childhood adversity or interpersonal trauma exhibited heightened avoidance, often mediated by shame, guilt, or fear (Feingold & Zerach, 2021; Gratz et al., 2007).

This pattern substantiates the hypothesis that avoidance functions as a short-term adaptive response to overwhelming affect, which—when chronically reinforced—transforms into a pathological regulation strategy. Such evidence corroborates neurobiological findings linking avoidance to dysregulated limbic activity and impaired prefrontal inhibition, which perpetuate craving and compulsive seeking behaviour.

### 4.3. Therapeutic and Clinical Implications

From a therapeutic perspective, the reviewed studies collectively advocate for the integration of third-wave behavioural approaches, particularly ACT, Dialectical Behaviour Therapy (DBT), and Mindfulness-Based Relapse Prevention (MBRP), as adjunctive or primary treatments for SUDs. These approaches target EA by fostering acceptance, emotional exposure, and value-oriented behaviour, thereby disrupting the avoidance–addiction cycle.

Empirical evidence across alcohol, tobacco, and polysubstance trials demonstrated that reductions in EA predicted improved treatment adherence, abstinence, and emotional stability (Cavicchioli et al., 2020; Luoma et al., 2020). Conversely, high baseline avoidance was consistently linked to poorer therapeutic outcomes and higher dropout rates (Hall et al., 2018; Stotts et al., 2015).

Clinically, EA should thus be considered both a treatment target and a predictor of outcome. Incorporating EA-sensitive measures, such as the Acceptance and Action Questionnaire-II (AAQ-II) or the Multidimensional Experiential Avoidance Questionnaire (MEAQ), into routine assessment can help identify patients at higher risk of relapse or disengagement. Moreover, training clinicians in acceptance-based exposure protocols can enhance intervention effectiveness across cultural and diagnostic boundaries.

### 4.4. Methodological Considerations and Limitations

In addition, legal status and regulatory regimes for psychoactive substances vary across countries; most included studies did not explicitly model policy context, so cross-country generalisation should be interpreted cautiously.

While the evidence base is strong, several limitations temper generalisability. The majority of studies were conducted in high-income Western countries, with limited representation from low- and middle-income contexts. Cross-sectional designs predominated, restricting causal inference, and the heterogeneity of instruments, particularly variations in EA operationalisation, introduced measurement variability. Additionally, few studies incorporated biological or longitudinal data, which would strengthen the causal chain linking EA, affect regulation, and relapse trajectories.

Nonetheless, methodological advances such as ecological momentary assessment (EMA) and multi-level modelling, employed in recent alcohol and tobacco research (Hooper et al., 2018; Martínez-Vispo et al., 2020), offer promising avenues for capturing the temporal dynamics of avoidance and craving in daily life. Future research should prioritise culturally adapted longitudinal designs and mixed-methods approaches integrating neurophysiological and self-report data.

### 4.5. Transdiagnostic Synthesis

When interpreted collectively, the findings establish EA as a transdiagnostic construct that transcends substance categories and diagnostic boundaries. Its consistent association with distress intolerance, affective avoidance, and maladaptive coping supports the view of addiction as a process of experiential control rather than merely a pharmacological dependence.

This conceptual shift has profound implications for prevention and intervention: rather than focusing solely on substance reduction, treatments should aim to cultivate psychological flexibility, enabling individuals to experience discomfort without resorting to escape behaviours. In this sense, EA represents both a diagnostic lens and a therapeutic lever for addressing the shared mechanisms underlying diverse forms of substance use.

## 5. Conclusions

This systematic review consolidates two decades of empirical evidence demonstrating that experiential avoidance (EA) is a core transdiagnostic process underpinning the development, persistence, and relapse of psychoactive substance use (PSU) across diverse populations and substance types.

From alcohol and tobacco to cannabis, cocaine, opioids, and polysubstance use, EA consistently emerged as a psychological mechanism of emotional regulation, whereby individuals seek to escape, suppress, or alter aversive internal states—such as anxiety, guilt, or traumatic memories—through substance consumption.

The cumulative findings indicate that EA not only predicts the severity of substance use and comorbid psychopathology but also influences treatment responsiveness and recovery trajectories. Individuals exhibiting high levels of avoidance are less likely to tolerate emotional discomfort during withdrawal, more prone to relapse, and less engaged in behavioural change processes. Conversely, interventions that foster acceptance, mindfulness, and psychological flexibility—particularly Acceptance and Commitment Therapy (ACT), Dialectical Behaviour Therapy (DBT), and Mindfulness-Based Relapse Prevention (MBRP)—demonstrate promising efficacy in reducing avoidance-driven behaviour and enhancing long-term recovery outcomes.

### 5.1. Conceptual Implications

The synthesis supports a paradigm shift in understanding addiction not merely as a behavioural or pharmacological disorder, but as a manifestation of experiential control—a rigid attempt to eliminate unwanted private experiences. EA provides an explanatory bridge between emotion regulation theories and third-wave behavioural models, integrating cognitive, affective, and motivational dimensions within a unified psychological framework. Importantly, the aim of psychological flexibility is consonant with long-standing cognitive–behavioural principles; third-wave approaches operationalise this aim through acceptance, defusion, and values-guided action (Hayes et al., 2006).

This conceptualisation underscores the need to view substance use through a process-based lens, emphasising the role of underlying mechanisms—such as avoidance and inflexibility—over symptom categories or substance types.

### 5.2. Methodological Implications

Methodologically, this review underscores the importance of standardising the assessment of EA using validated and psychometrically robust tools, such as the Acceptance and Action Questionnaire-II (AAQ-II) and the Multidimensional Experiential Avoidance Questionnaire (MEAQ).

The predominance of cross-sectional designs limits causal inference, calling for longitudinal and experimental studies that can disentangle temporal relationships between avoidance, emotional regulation, and substance use. Future research should also integrate neurobiological and ecological momentary assessment (EMA) data to elucidate the real-time dynamics of avoidance and craving.

Additionally, expanding research beyond high-income Western contexts is essential to capture sociocultural variability in avoidance processes and treatment responsiveness.

### 5.3. Clinical Implications

Clinically, EA should be regarded as both a diagnostic marker and a treatment target within addiction interventions. Screening for avoidance-related tendencies can help clinicians identify individuals at greater risk of relapse, while tailoring interventions to cultivate acceptance, emotional exposure, and value-based action can foster resilience and sustainable recovery.

Integrating ACT principles within standard substance use treatment protocols could improve motivation for change, emotional stability, and self-regulation, while decreasing avoidance behaviours that perpetuate dependence. The inclusion of EA-informed frameworks within public health strategies may thus enhance the efficiency and precision of addiction prevention and intervention programmes.

### 5.4. Final Reflection

In conclusion, the reviewed evidence positions experiential avoidance as a central psychological mechanism linking affective distress and addictive behaviour across substance categories and clinical profiles. Its role as a transdiagnostic factor redefines the conceptual boundaries of addiction science, shifting focus from the substance itself to the functional processes that maintain maladaptive coping.

Future work should deepen this integrative approach by examining the biopsychosocial interplay between avoidance, emotion regulation, and neurocognitive functioning, paving the way for process-based, personalised treatment models that transcend traditional diagnostic silos.

## Figures and Tables

**Table 1:** PICOS structure of the research question.

Component	Description
Population (P)	Clinical and non-clinical populations (adolescents and adults) who report psychoactive substance use (alcohol, tobacco/nicotine, cannabis, cocaine, opioids, or other illicit substances, including polysubstance use).
Intervention/Exposure (I/E)	Experiential avoidance/psychological inflexibility assessed with validated instruments (e.g., AAQ-II, MEAQ, AAQ-SA) or clearly defined operationalisations within ACT/third-wave frameworks.
Comparator (C)	Lower EA/greater psychological flexibility, or study-defined between-group contrasts (e.g., low vs. high EA; treatment responders vs. non-responders; pre vs. post intervention).
Outcomes (O)	Substance use behaviour and severity (frequency/quantity, craving, dependence symptoms, relapse/abstinence) and treatment outcomes; secondary outcomes included emotional and behavioural correlates linked to EA.
Study design (S)	Quantitative observational (cross-sectional, longitudinal) and experimental/quasi-experimental designs.

**Table 2:** DeCS and MeSH descriptors used in the search.

Term Category	Descriptors Used
Experiential Avoidance	“Experiential Avoidance”, “Psychological Inflexibility”, “Avoidance Coping”, “Acceptance and Commitment Therapy”
Substance Use	“Substance Use Disorders”, “Substance Dependence”, “Alcohol Abuse”, “Drug Abuse”, “Substance Abuse”, “Addiction”, “Psychoactive Substance Use”
Population	“Adults”, “Adolescents”, “Clinical Populations”, “Non-Clinical Populations”, “Patients”, “Community Samples”

**Table 3:** Search algorithms applied in each database.

Database	Search Algorithm
SCOPUS	TITLE-ABS-KEY (“Experiential Avoidance” AND (“Substance Use Disorders” OR “Substance Dependence” OR “Alcohol Abuse” OR “Drug Abuse” OR “Substance Use” OR “Addiction”))
PubMed	(“Experiential Avoidance”[All Fields]) AND (“Substance Use Disorders”[MeSH Terms] OR “Substance Dependence” OR “Alcohol Abuse” OR “Drug Abuse” OR “Substance Use”)
Web of Science	ALL = (“Experiential Avoidance” AND (“Substance Use Disorders” OR “Substance Dependence” OR “Alcohol Abuse” OR “Drug Abuse” OR “Substance Abuse” OR “Addiction”))
APA PsycNet	(“Experiential Avoidance”) AND (“Substance Use” OR “Addiction” OR “Substance Abuse”)

**Table 4:** Database contribution to records identified, deduplication, screening, and included studies.

Database	Records Identified (*n*)	Duplicate Records Removed (*n*)	Records Screened After Deduplication (*n*)	Included Studies (*n*)
SCOPUS	78	23	55	12
PubMed	102	30	72	12
Web of Science	217	95	122	10
APA PsycNet	34	11	23	6
Additional sources (citation tracking)	1	0	1	1
Total	432	159	273	41

**Table 5:** Summary of included studies by type of psychoactive substance.

ID	Authors (Year)	Country	Substance	Study Design	Sample	Instruments/Procedure	Key Variables	Key Findings
1	Luoma et al. (2020)	USA	Alcohol	Cross-sectional (meta-analysis of RCT data)	193 young adults (risk drinkers)	DASS; UPPS-P; DDQ-R; YAACQ; DMQ	Alcohol problems, anxiety, impulsivity, coping motives	Anxiety related to physiological dependence; negative urgency linked to dysregulated behaviour and coping-motivated drinking.
2	Luoma et al. (2020)	USA	Alcohol	Cross-sectional (daily EMA)	206 adults	PANAS; daily AAQ; AAQ-II; AUDIT	Experiential avoidance; drinking alone	Higher avoidance and negative affect predicted drinking alone more often, and higher affect linked to greater intake.
3	Levin et al. (2012)	USA	Alcohol	Cross-sectional correlational	240 college students	SCID; RAPI; AAQ-II; GHQ	Experiential avoidance; alcohol use disorder	Students with alcohol abuse/dependence showed higher experiential avoidance than non-dependent peers.
4	Vernig and Orsillo (2009)	USA	Alcohol	Analogue (pre–post)	48 students	Exposure to aversive images; SAM; AUDIT; DAST-10; coping questionnaire	Mindfulness, avoidance, reactivity	Mindfulness group showed better coping; suggests drinking as emotion regulation.
5	Klanecky et al. (2012)	USA	Alcohol	Cross-sectional correlational	298 students	AUDIT; DES-II; DDS; ETISR-SF	Childhood abuse, dissociation, alcohol problems	Childhood sexual abuse correlated with problematic drinking and experiential avoidance, though not direct mediation.
6	Feingold and Zerach (2021)	Israel	Alcohol	Correlational	189 combat veterans (men)	CES; PCL-5; AUDIT; ERQ; AAQ-II	PTSD, avoidance, emotion regulation	PTSD linked to AUD, higher avoidance/suppression, lower reappraisal.
7	Sheynin et al. (2019)	Australia	Alcohol	Experimental pilot	Men with dependence vs. controls	Computer task measuring approach/avoidance	Approach, avoidance, escape	Alcohol-dependent patients showed more avoidance and escape behaviours.
8	E. S. Lee and Bong (2018)	South Korea	Alcohol	Cross-sectional (SEM)	383 students	CTQ-SF; CAST; CES-D; AAQ-II; SSI	Parental alcoholism, trauma, avoidance, depression	Parental alcohol use → trauma → avoidance/depression → suicidal ideation.
9	Cavicchioli et al. (2020)	Italy	Alcohol	Experimental (DBT feasibility)	171 outpatients	DBT training (36 sessions); ASI; DERS; AAQ-II	AUD, emotion dysregulation, avoidance	DBT reduced avoidance and emotion dysregulation, supporting abstinence.
10	Dvorak et al. (2013)	USA	Alcohol	Correlational (path analysis)	313 trauma-exposed students	AUDIT; MEAQ; PTSD scale	Distress tolerance/aversion, trauma, alcohol use	High PTSD: tolerance protective; low PTSD: aversion predictive of problems.
11	Martínez-Vispo et al. (2020)	Spain	Tobacco	Cross-sectional (serial mediation)	275 treatment-seeking smokers	BADS; Fagerström; EROS; BDI-II	Avoidance, depression, reward, dependence	Avoidance linked to depression and low reward; both predicted higher dependence.
12	Mak et al. (2021)	Hong Kong	Tobacco	RCT (pilot)	65 smokers with schizophrenia	12-week ACT vs. support; AAQ; regulation scales	ACT, avoidance, regulation, abstinence	ACT reduced avoidance/regulation issues but not quit rates (small *n*).
13	Hooper et al. (2018)	UK	Tobacco	Experimental	54 student smokers	3 conditions: defusion, avoidance, control	Defusion, motivation	Defusion boosted motivation to quit more than avoidance or control.
14	Farris et al. (2016a)	USA	Tobacco	Clinical trial (pre–post)	149 smokers	Standard vs. anxiety-focused; AIS	Abstinence, avoidance	Abstainers reported lower post-quit avoidance; women had higher baseline avoidance.
15	Farris et al. (2016b)	USA	Tobacco	Cross-sectional	465 daily smokers	SCID; FTND; PANAS; PSWQ; AIS	Worry, dysphoria, avoidance	Trait worry increased smoking-specific avoidance → greater barriers and motives.
16	Watson et al. (2017)	USA	Tobacco	Cross-sectional	450 smokers	Mini-SPIN; AIS; FTND; CES-D	Social anxiety, avoidance	Social anxiety linked to greater avoidance of internal smoking cues.
17	Bakhshaie et al. (2016)	USA	Tobacco	Cross-sectional	217 trauma-exposed smokers	SHQ; FTND; CO; AUDIT; ASI-3; AIS	Anxiety sensitivity; avoidance	Anxiety sensitivity predicted smoking severity via avoidance.
18	Karekla et al. (2017)	Cyprus	Tobacco	Laboratory	35 smokers (8 h abstinence)	PASAT-C; HR; SCL; EMG	Stress, craving, affect	Stress induction increased craving and negative affect.
19	Garey et al. (2016)	USA	Tobacco	Cross-sectional	448 smokers	ASI-3; IDAS; SCQ; AUDIT; BFI	Anxiety, dysphoria, avoidance	Anxiety/dysphoria related to avoidance and maladaptive smoking cognitions.
20	Robles et al. (2016)	USA	Tobacco	Cross-sectional (RCT baseline)	332 smokers	PSS; SCQ-NA; AIS; PANAS	Stress, avoidance, barriers	Stress and avoidance linked to barriers and failure in prior quit attempts.
21	Jardin et al. (2015)	USA	Tobacco	Cross-sectional	396 smokers	SCID; FTND; CO; ASI-3; AIS	Anxiety sensitivity; dependence	Greater sensitivity/avoidance predicted higher nicotine dependence.
22	Buckner and Zvolensky (2014)	USA	Cannabis	Correlational	123 cannabis users	MCSAS; MMM; MEEQ; MEAQ	Cannabis use; social anxiety; avoidance	Cannabis use to manage social anxiety reinforced dependence.
23	Twohig et al. (2007)	USA	Cannabis	Multiple-case pilot	3 adults with dependence	8 ACT sessions; BAI; BDI-II; AAQ	ACT, avoidance, abstinence	ACT promoted abstinence; one brief relapse; pilot evidence.
24	Stotts et al. (2015)	USA	Cocaine	Post hoc analysis	99 outpatients	CM programme; AIS; BDI-II; DASS-21	Avoidance, CM outcome	High avoidance predicted poor abstinence under CM.
25	Naifeh et al. (2012)	USA	Cocaine	Cross-sectional	62 inpatients (crack/cocaine + trauma)	CAPS; ASI; EAQ; DASS	Emotional avoidance, PTSD	Avoidance and anxiety sensitivity associated with greater PTSD severity.
26	Serowik and Orsillo (2019)	USA	Multiple	Cross-sectional	223 students	RAPI; DAST-10; DMQ; MEAQ	Avoidance, substance use, values	Higher value importance → fewer alcohol/drug problems; coping motives predicted use.
27	Brem et al. (2017a)	USA	Multiple	Cross-sectional (retrospective)	150 men (residential SUD)	AAQ-II; PDSQ; SAST-R; AUDIT	Avoidance, depression, CSB	Mixed results: distress/CSB positively related; avoidance inconsistently related.
28	Elmquist et al. (2018)	USA	Multiple	Exploratory regression	108 men (residential SUD)	AUDIT; DUDIT; PDSQ; AAQ-II	Avoidance, bulimia, SUD	Avoidance associated with both bulimia and substance problems.
29	Forsyth et al. (2003)	USA	Multiple	Correlational (pre–post)	94 veterans	ASI; BSQ; ACQ; AAQ; BDI	Anxiety, avoidance, control	Higher reactivity/depression linked to greater avoidance and low control.
30	Aurora and Coifman (2021)	USA	Multiple	Diary study	195 students (social anxiety)	Mini-SPIN; Big-5; daily logs	Social anxiety, extraversion, use	Extraversion moderated coping: extroverts used substances socially; introverts avoided situations.
31	Fowler et al. (2016)	USA	Multiple	Prospective inpatient	994 psychiatric patients	AAQ-II; DERS; SCID	Avoidance, emotion regulation, SUD	Avoidance and dysregulation improved during admission; greater improvement among SUD patients.
32	Baker et al. (2007)	Australia	Multiple	Pilot	24 rehabilitation inpatients	Group music therapy (7 × 1.5 h)	Emotion regulation, avoidance	87.5% reported positive mood change; preliminary evidence.
33	Brem et al. (2017b)	USA	Multiple	Correlational (mediation)	175 men (residential SUD)	MAAS; AAQ-II; SAST-R; AUDIT; DUDIT	Mindfulness, avoidance, CSB	Mindfulness reduced avoidance, which in turn reduced CSB and substance use.
34	Gratz et al. (2007)	USA	Multiple	Laboratory correlational	76 patients	CTQ-SF; DERS; DSM-IV interview	Child abuse, avoidance, non-acceptance	Severe abuse predicted higher avoidance and low emotional acceptance.
35	Brem et al. (2018)	USA	Multiple	Cross-sectional	446 women (residential SUD)	AAQ-II; YSQ-L3; PDSQ; SAST-R; AUDIT; DUDIT	Shame, PTSD, CSB, avoidance	Avoidance mediated between PTSD/shame and CSB; addressing both may improve outcomes.
36	Hall et al. (2018)	Australia	Multiple	Single-arm pilot	45 adults (SUD + BPD)	ACT (12 sessions); DERS; AAQ-II	BPD, avoidance, dysregulation	ACT reduced use, avoidance, and dysregulation; high dropout rate.
37	Chapman and Cellucci (2007)	USA	Multiple	Cross-sectional	117 incarcerated women	SCID-II; CTQ; BDI-II; AAQ; WBSI	BPD/ASPD, avoidance, cognition	Personality traits linked to dependence; thought suppression key predictor.
38	Kingston et al. (2010)	UK	Multiple	Cross-sectional (SEM)	290 participants	AIM-NI; CTQ-SF; CMPB; AAQ; WBSI	Avoidance, trauma, affect intensity	Avoidance mediated trauma/affect → risky behaviour; overlap with dysregulation.
39	Chartier et al. (2010)	USA	Multiple	Correlational	23 gay men (HIV + meth use)	AAQ-II; THQ; PCL-C; MCIS	Avoidance, trauma, illness management	Longer HIV diagnosis linked to more PTSD; integrate trauma and illness care.
40	Fazeli Rad et al. (2024)	Iran	Multiple	Cross-sectional	241 men (opioid treatment ≥ 8 yrs)	DTS; DDQ; AAQ-II; DERS	Craving, avoidance, distress tolerance	Avoidance mediated distress tolerance → craving; improve tolerance and acceptance in therapy.
41	Ulusoy et al. (2022)	Turkiye	Multiple substances (SUD)	Cross-sectional (mediation analysis)	111 patients with substance use disorders	AAQ-SA, BDI, Addiction Profile Index (API), and Forms of Self-Criticizing/Attacking and Self-Reassuring Scale (FSCRS). Mediation tested using regression-based path analysis with bootstrap.	Psychological inflexibility and self-criticism mediated the association between depressive symptoms and addiction severity.	Depressive symptoms were linked to greater addiction severity; psychological inflexibility and self-criticism mediated this association in patients with substance use disorders.

Abbreviations: AAQ-II: *Acceptance and Action Questionnaire-II*; AIS: *Avoidance and Inflexibility Scale*; ASI-3: *Anxiety Sensitivity Index-3*; AUDIT: *Alcohol Use Disorders Identification Test*; BDI-II: *Beck Depression Inventory-II*; CTQ-SF: *Childhood Trauma Questionnaire–Short Form*; DASS: *Depression, Anxiety and Stress Scales*; DERS: *Difficulties in Emotion Regulation Scale*; EMA: *Ecological Momentary Assessment*; ERQ: *Emotion Regulation Questionnaire*; FTND: *Fagerström Test for Nicotine Dependence*; MEAQ: *Multidimensional Experiential Avoidance Questionnaire*; PANAS: *Positive and Negative Affect Schedule*; PCL: *PTSD Checklist*; SCID: *Structured Clinical Interview for DSM*; PTSD: *Post-Traumatic Stress Disorder*; AUD/SUD: *Alcohol/Substance Use Disorder*; CSB: *Compulsive Sexual Behaviour*; ACT: *Acceptance and Commitment Therapy*; DBT: *Dialectical Behaviour Therapy*.

**Table 6:** General characteristics of included studies (*n* = 41).

Dimension	Summary Distribution
Geographic distribution	USA (*n* = 28, ~68%); Europe (*n* = 5, ~12%); Brazil (*n* = 3, ~7%); Asia (*n* = 4, ~10%; including Turkey); and Israel (*n* = 1, ~2%).
Study design	Cross-sectional/correlational (~60%); experimental/laboratory (~15%); clinical pre–post/trial (~15%); pilot (~7%); diary/prospective (~3%)
Populations	Students; treatment samples (outpatient/residential); smokers; veterans; individuals with psychiatric comorbidities; community samples
Substance types	Tobacco (*n* = 10, ~24.4%); alcohol (*n* = 11, ~26.8%); cannabis (*n* = 2, ~4.9%); cocaine (*n* = 2, ~4.9%); opiate (*n* = 1, ~2.4%); and multiple substances (*n* = 15, ~36.6%).
EA measures	AAQ-II, MEAQ, AIS
Other measures	DERS, PANAS, CES-D, BDI-II, ASI-3, AUDIT, FTND, DUDIT
Therapeutic approaches	ACT, DBT, cognitive defusion, music therapy, contingency management
General trends	EA was positively associated with substance-use severity, emotional dysregulation, anxiety, coping-related motives, craving, and relapse barriers. ACT and DBT interventions reduced EA and improved emotion regulation.

**Table 7:** Alcohol: Variables, methods, and key findings (IDs 1–10).

ID	Design	Sample	Main Measures	Analytical Focus	Main Findings	Reference
1	Cross-sectional (meta-analysis of RCT data)	193 young adult risk drinkers	DASS, UPPS-P, DDQ-R, YAACQ, DMQ	Anxiety, negative urgency, coping motives	Anxiety was associated with physiological dependence; negative urgency predicted dysregulated and coping-driven drinking	Luoma et al. (2020)
2	Cross-sectional (daily EMA)	206 adults	PANAS, daily AAQ, AAQ-II, AUDIT	Daily avoidance and affect	Higher EA and negative affect predicted greater likelihood of solitary drinking and higher alcohol intake	Luoma et al. (2020)
3	Correlational	240 students	SCID, RAPI, AAQ-II, GHQ	Alcohol use disorder diagnosis	Students with alcohol abuse or dependence showed higher EA than non-dependent peers	Levin et al. (2012)
4	Analogue (pre–post)	48 students	SAM, AUDIT, DAST-10, coping questionnaire	Mindfulness versus control	The mindfulness group demonstrated better coping and lower avoidance than controls	Vernig and Orsillo (2009)
5	Correlational	298 students	AUDIT, DES-II, DDS, ETISR-SF	Childhood abuse, dissociation, EA	Childhood sexual abuse was associated with both higher experiential avoidance and greater alcohol-related problems	Klanecky et al. (2012)
6	Correlational	189 veterans	CES, PCL-5, AUDIT, ERQ, AAQ-II	PTSD and emotion regulation	PTSD symptoms were associated with alcohol use disorder, higher EA, increased suppression, and lower reappraisal	Feingold and Zerach (2021)
7	Experimental pilot	Men with alcohol dependence and controls	Computer task (approach/avoidance/escape)	Motivational bias	Dependent participants exhibited stronger avoidance and escape behaviours	Sheynin et al. (2019)
8	SEM	383 students	CTQ-SF, CAST, CES-D, AAQ-II, SSI	Parental alcoholism, trauma, depression	Parental drinking predicted trauma, which in turn predicted avoidance and depression leading to suicidal ideation	E. S. Lee and Bong (2018)
9	Experimental (DBT)	171 outpatients with alcohol use disorder	DBT (36 sessions), ASI, DERS, AAQ-II	Emotional regulation and abstinence	Participants showed reduced avoidance and emotional dysregulation, supporting abstinence	Cavicchioli et al. (2020)
10	Correlational (path analysis)	313 trauma-exposed students	AUDIT, MEAQ, PTSD scale	Distress tolerance and aversion	Experiential avoidance mediated the relationship between PTSD and alcohol problems	Dvorak et al. (2013)

**Table 8:** Tobacco: Variables, methods, and key findings (IDs 11–21).

ID	Design	Sample	Main Measures	Analytical Focus	Main Findings	Reference
11	Cross-sectional (serial mediation)	275 treatment-seeking smokers	BADS, Fagerström, EROS, BDI-II	Avoidance, depression, reward	Avoidance was associated with higher depression and lower reward, both predicting stronger nicotine dependence	Martínez-Vispo et al. (2020)
12	RCT (pilot)	65 smokers with schizophrenia	ACT (12 weeks), AAQ, regulation scales	ACT vs. support therapy	ACT reduced avoidance and improved emotion regulation, but abstinence rates did not differ significantly	Mak et al. (2021)
13	Experimental	54 student smokers	Defusion, avoidance, control conditions	Defusion and motivation	Cognitive defusion increased motivation to quit compared with avoidance or control conditions	Hooper et al. (2018)
14	Clinical trial (pre–post)	149 smokers	Standard vs. anxiety-focused, AIS	Abstinence, avoidance	Successful abstainers showed lower post-quit EA; women exhibited higher baseline EA	Farris et al. (2016a)
15	Cross-sectional	465 daily smokers	SCID, FTND, PANAS, PSWQ, AIS	Worry, dysphoria, avoidance	Trait worry and dysphoria were indirectly linked to smoking through higher EA	Farris et al. (2016b)
16	Cross-sectional	450 smokers	Mini-SPIN, AIS, FTND, CES-D	Social anxiety, EA	Social anxiety was associated with higher smoking-specific EA and greater difficulty resisting cravingayuds	Watson et al. (2017)
17	Cross-sectional	217 trauma-exposed smokers	SHQ, FTND, CO, AUDIT, ASI-3, AIS	Anxiety sensitivity, avoidance	Anxiety sensitivity predicted smoking severity through avoidance	Bakhshaie et al. (2016)
18	Laboratory	35 smokers (8 h abstinence)	PASAT-C, HR, SCL, EMG	Stress induction and craving	Acute stress increased craving and negative affect, suggesting avoidance-based regulation	Karekla et al. (2017)
19	Cross-sectional	448 smokers	ASI-3, IDAS, SCQ, AUDIT, BFI	Anxiety, dysphoria, avoidance	Anxiety and dysphoria were linked to maladaptive smoking cognitions mediated by avoidance	Garey et al. (2016)
20	Cross-sectional (RCT baseline)	332 smokers	PSS, SCQ-NA, AIS, PANAS	Stress, avoidance, relapse barriers	Stress and avoidance were associated with increased perceived barriers and prior quit failures	Robles et al. (2016)
21	Cross-sectional	396 smokers	SCID, FTND, CO, ASI-3, AIS	Anxiety sensitivity and dependence	Higher anxiety sensitivity and avoidance predicted greater nicotine dependence	Jardin et al. (2015)

**Table 9:** Cannabis: Variables, methods, and key findings (IDs 22–23).

ID	Design	Sample	Main Measures	Analytical Focus	Main Findings	Reference
22	Correlational	123 cannabis users	MCSAS, MMM, MEEQ, MEAQ	Social anxiety and EA	High social anxiety was associated with cannabis use to avoid negative internal states and greater dependence severity	Buckner and Zvolensky (2014)
23	Multiple-case pilot	3 adults with cannabis dependence	ACT sessions (8), BAI, BDI-II, AAQ	EA and treatment response	ACT reduced EA and supported abstinence in most cases; one short relapse was observed	Twohig et al. (2007)

**Table 10:** Cocaine: Variables, methods, and key findings (IDs 24–25).

ID	Design	Sample	Main Measures	Analytical Focus	Main Findings	Reference
24	Post hoc analysis	99 outpatients in CM programme	AIS, BDI-II, DASS-21	Avoidance and abstinence outcomes	Higher avoidance predicted lower abstinence rates and poorer CM treatment response	Stotts et al. (2015)
25	Cross-sectional	62 inpatients (crack/cocaine + trauma)	CAPS, ASI, EAQ, DASS	Emotional avoidance, PTSD, anxiety sensitivity	Avoidance and anxiety sensitivity were associated with higher PTSD severity and substance use	Naifeh et al. (2012)

**Table 11:** Opioids: Variables, methods, and key findings (ID 40).

ID	Design	Sample	Main Measures	Analytical Focus	Main Findings	Reference
40	Cross-sectional	241 men in opioid treatment (≥8 years)	DTS, DDQ, AAQ-II, DERS	Distress tolerance, craving, avoidance	EA mediated the relationship between distress tolerance and craving; enhancing acceptance and tolerance may improve recovery outcomes	Fazeli Rad et al. (2024)

**Table 12:** Polysubstance and multiple substance use: Variables, methods, and key findings (IDs 26–39).

ID	Design	Sample	Main Measures	Analytical Focus	Main Findings	Reference
26	Cross-sectional	223 students	RAPI, DAST-10, DMQ, MEAQ	Avoidance, values, substance use	Higher avoidance and coping motives predicted more problems; value orientation was protective	Serowik and Orsillo (2019)
27	Cross-sectional (retrospective)	150 men in residential SUD	AAQ-II, PDSQ, SAST-R, AUDIT	Avoidance, depression, compulsive sexual behaviour	Avoidance correlated with distress and compulsive sexual behaviour, but effects were inconsistent	Brem et al. (2017a)
28	Exploratory regression	108 men in residential SUD	AUDIT, DUDIT, PDSQ, AAQ-II	Avoidance, bulimia, SUD	EA associated with bulimic symptoms and SUD severity	Elmquist et al. (2018)
29	Correlational (pre–post)	94 veterans	ASI, BSQ, ACQ, AAQ, BDI	Anxiety, avoidance, control	Depression and reactivity predicted high avoidance and low control	Forsyth et al. (2003)
30	Diary study	195 students (social anxiety)	Mini-SPIN, Big-5, daily logs	Social anxiety, extraversion, use	Extraversion moderated avoidance and social use; introverts used substances to avoid exposure	Aurora and Coifman (2021)
31	Prospective inpatient	994 psychiatric patients	AAQ-II, DERS, SCID	Avoidance, emotion regulation	Avoidance and dysregulation improved during treatment; largest effect among SUD patients	Fowler et al. (2016)
32	Pilot intervention	24 inpatients (rehabilitation)	Music therapy sessions	Emotion regulation, avoidance	87.5% reported mood improvement; experiential engagement reduced avoidance	Baker et al. (2007)
33	Correlational (mediation)	175 men in residential SUD	MAAS, AAQ-II, SAST-R, AUDIT, DUDIT	Mindfulness, avoidance, compulsive sexual behaviour	Mindfulness reduced avoidance, which mediated lower substance and CSB levels	Brem et al. (2017b)
34	Laboratory correlational	76 patients	CTQ-SF, DERS, DSM-IV interview	Childhood abuse, avoidance	Abuse severity predicted high avoidance and poor emotional acceptance	Gratz et al. (2007)
35	Cross-sectional	446 women (residential SUD)	AAQ-II, YSQ-L3, PDSQ, SAST-R, AUDIT, DUDIT	Shame, PTSD, avoidance	Avoidance mediated the relationship between shame/PTSD and compulsive sexual behaviour	Brem et al. (2018)
36	Single-arm pilot	45 adults (SUD + BPD)	ACT sessions, DERS, AAQ-II	BPD, avoidance, emotion regulation	ACT reduced avoidance and emotional dysregulation; dropout limited generalisability	Hall et al. (2018)
37	Cross-sectional	117 incarcerated women	SCID-II, CTQ, BDI-II, AAQ, WBSI	Personality traits, avoidance	BPD/ASPD traits predicted substance dependence via thought suppression and avoidance	Chapman and Cellucci (2007)
38	Cross-sectional (SEM)	290 participants	AIM-NI, CTQ-SF, CMPB, AAQ, WBSI	Trauma, affect intensity, avoidance	Avoidance mediated trauma–affect–risk pathway; key role in risky behaviour	Kingston et al. (2010)
39	Correlational	23 MSM with HIV using methamphetamine	AAQ-II, THQ, PCL-C, MCIS	Trauma, illness management, avoidance	PTSD symptoms and avoidance increased with illness duration; recommend trauma-integrated care	Chartier et al. (2010)

## Data Availability

Data sharing is not applicable to this article, as it is based exclusively on previously published studies. No new datasets were generated or analysed in this systematic review.

## Supplementary Materials

The following supporting information can be downloaded at: https://www.mdpi.com/article/10.3390/ejihpe16020022/s1, PRISMA 2020 Checklist.

## List of Abbreviations

Abbreviation	Full Term
AAQ-II	Acceptance and Action Questionnaire-II
ACT	Acceptance and Commitment Therapy
AIS	Avoidance and Inflexibility Scale
APA	American Psychological Association
ASI-3	Anxiety Sensitivity Index-3
AUDIT	Alcohol Use Disorders Identification Test
BADS	Behavioural Activation for Depression Scale
BDI-II	Beck Depression Inventory-II
CTQ-SF	Childhood Trauma Questionnaire–Short Form
DASS	Depression, Anxiety and Stress Scales
DBT	Dialectical Behaviour Therapy
DERS	Difficulties in Emotion Regulation Scale
EMA	Ecological Momentary Assessment
ERQ	Emotion Regulation Questionnaire
FTND	Fagerström Test for Nicotine Dependence
MEAQ	Multidimensional Experiential Avoidance Questionnaire
PANAS	Positive and Negative Affect Schedule
PCL-5	PTSD Checklist for DSM-5
PRISMA	Preferred Reporting Items for Systematic Reviews and Meta-Analyses
PSU	Psychoactive Substance Use
PTSD	Post-Traumatic Stress Disorder
SUD	Substance Use Disorder
WHO	World Health Organization

## References

[B1-ejihpe-16-00022] Aurora P., Coifman K. G. (2021). Unpacking social avoidance and substance use in social anxiety: Does extraversion explain behavior variability?. Journal of Psychopathology and Behavioral Assessment.

[B2-ejihpe-16-00022] Baker F. A., Gleadhill L. M., Dingle G. A. (2007). Music therapy and emotional exploration: Exposing substance abuse clients to the experiences of non-drug-induced emotions. Arts in Psychotherapy.

[B3-ejihpe-16-00022] Bakhshaie J., Zvolensky M. J., Salazar A., Vujanovic A. A., Schmidt N. B. (2016). Anxiety sensitivity and smoking behavior among trauma-exposed daily smokers: The explanatory role of smoking-related avoidance and inflexibility. Behavior Modification.

[B4-ejihpe-16-00022] Barrado-Moreno V., Serrano-Ibáñez E. R., Esteve R., Ramírez-Maestre C., Sánchez-Meca J. (2025). The role of psychological flexibility and inflexibility in substance addiction, abuse, or misuse: A systematic review and meta-analysis. International Journal of Mental Health and Addiction.

[B5-ejihpe-16-00022] Brem M. J., Shorey R. C., Anderson S., Stuart G. L. (2017a). Depression, anxiety, and compulsive sexual behaviour among men in residential treatment for substance use disorders: The role of experiential avoidance. Clinical Psychology and Psychotherapy.

[B6-ejihpe-16-00022] Brem M. J., Shorey R. C., Anderson S., Stuart G. L. (2017b). Experiential avoidance as a mediator of the relationship between dispositional mindfulness and compulsive sexual behaviors among men in residential substance use treatment. Sexual Addiction and Compulsivity.

[B7-ejihpe-16-00022] Brem M. J., Shorey R. C., Anderson S., Stuart G. L. (2018). Does experiential avoidance explain the relationships between shame, PTSD symptoms, and compulsive sexual behaviour among women in substance use treatment?. Clinical Psychology and Psychotherapy.

[B8-ejihpe-16-00022] Buckner J. D., Zvolensky M. J. (2014). Cannabis and related impairment: The unique roles of cannabis use to cope with social anxiety and social avoidance. American Journal on Addictions.

[B9-ejihpe-16-00022] Buckner J. D., Zvolensky M. J., Farris S. G., Hogan J. (2014). Social anxiety and coping motives for cannabis use: The impact of experiential avoidance. Psychology of Addictive Behaviors.

[B10-ejihpe-16-00022] Cavicchioli M., Movalli M., Ramella P., Vassena G., Prudenziati F., Maffei C. (2020). Feasibility of dialectical behavior therapy skills training as an outpatient program in treating alcohol use disorder: The role of difficulties with emotion regulation and experiential avoidance. Addiction Research and Theory.

[B11-ejihpe-16-00022] Chapman A. L., Cellucci T. (2007). The role of antisocial and borderline personality features in substance dependence among incarcerated females. Addictive Behaviors.

[B12-ejihpe-16-00022] Chartier M., Vinatieri T., DeLonga K., McGlynn L. M., Gore-Felton C., Koopman C. (2010). A pilot study investigating the effects of trauma, experiential avoidance, and disease management in HIV-positive MSM using methamphetamine. Journal of the International Association of Physicians in AIDS Care.

[B13-ejihpe-16-00022] Dvorak R. D., Arens A. M., Kuvaas N. J., Williams T. J., Kilwein T. M. (2013). Problematic alcohol use, trauma history, and ptsd symptom level: A path analysis. Journal of Dual Diagnosis.

[B14-ejihpe-16-00022] Ehman A. C., Gross A. M. (2019). Acceptance and commitment therapy and motivational interviewing in the treatment of alcohol use disorder in a college woman: A case study. Clinical Case Studies.

[B15-ejihpe-16-00022] Elmquist J. A., Shorey R. C., Anderson S., Stuart G. L. (2018). Experiential avoidance and bulimic symptoms among men in residential treatment for substance use disorders: A preliminary examination. Journal of Psychoactive Drugs.

[B16-ejihpe-16-00022] Farris S. G., Dibello A. M., Heggeness L. F., Reitzel L. R., Vidrine D. J., Schmidt N. B., Zvolensky M. J. (2016a). Sustained smoking abstinence is associated with reductions in smoking-specific experiential avoidance among treatment-seeking smokers. Journal of Behavior Therapy and Experimental Psychiatry.

[B17-ejihpe-16-00022] Farris S. G., Zvolensky M. J., Norton P. J., Hogan J., Smith A. H., Talkovsky A. M., Garey L., Schmidt N. B. (2016b). Smoking-specific experiential avoidance is indirectly associated with trait worry and smoking processes among treatment-seeking smokers. Behavioral Medicine.

[B18-ejihpe-16-00022] Fazeli Rad H., Noury Ghaesm Abadi R., Hasani J. (2024). Craving, distress tolerance, emotion dysregulation, and experiential avoidance among patients in early recovery from opioid use disorder in residential programs. Journal of Substance Use.

[B19-ejihpe-16-00022] Feingold D., Zerach G. (2021). Emotion regulation and experiential avoidance moderate the association between posttraumatic symptoms and alcohol use disorder among Israeli combat veterans. Addictive Behaviors.

[B20-ejihpe-16-00022] Forsyth J. P., Parker J. D., Finlay C. G. (2003). Anxiety sensitivity, controllability, and experiential avoidance and their relation to drug of choice and addiction severity in a residential sample of substance-abusing veterans. Addictive Behaviors.

[B21-ejihpe-16-00022] Fowler J. C., Clapp J. D., Madan A., Allen J. G., Oldham J. M., Frueh B. C. (2016). Emotion dysregulation as a cross-cutting target for inpatient psychiatric intervention. Journal of Affective Disorders.

[B22-ejihpe-16-00022] Garey L., Farris S. G., Schmidt N. B., Zvolensky M. J. (2016). The role of smoking-specific experiential avoidance in the relation between perceived stress and tobacco dependence, perceived barriers to cessation, and problems during quit attempts among treatment-seeking smokers. Journal of Contextual Behavioral Science.

[B23-ejihpe-16-00022] Garland E. L., Gaylord S. A., Boettiger C. A., Howard M. O. (2010). Mindfulness training modifies cognitive, affective, and physiological mechanisms implicated in alcohol dependence: Results of a randomized controlled pilot trial. Journal of Psychoactive Drugs.

[B24-ejihpe-16-00022] Garland E. L., Howard M. O. (2018). Mindfulness-based treatment of addiction: Current state of the field and envisioning the next wave of research. Addiction Science and Clinical Practice.

[B25-ejihpe-16-00022] Grant S., Colaiaco B., Motala A., Shanman R., Booth M., Sorbero M., Hempel S. (2017). Mindfulness-based relapse prevention for substance use disorders: A systematic review and meta-analysis. Journal of Addiction Medicine.

[B26-ejihpe-16-00022] Gratz K. L., Bornovalova M. A., Delany-Brumsey A., Nick B., Lejuez C. W. (2007). A laboratory-based study of the relationship between childhood abuse and experiential avoidance among inner-city substance users: The role of emotional nonacceptance. Behavior Therapy.

[B27-ejihpe-16-00022] Hall K., Simpson A., O’Donnell R., Sloan E., Staiger P. K., Morton J., Ryan D., Nunn B., Best D., Lubman D. I. (2018). Emotional dysregulation as a target in the treatment of co-existing substance use and borderline personality disorders: A pilot study. Clinical Psychologist.

[B28-ejihpe-16-00022] Hayes S. C., Luoma J. B., Bond F. W., Masuda A., Lillis J. (2006). Acceptance and commitment therapy: Model, processes and outcomes. Behaviour Research and Therapy.

[B29-ejihpe-16-00022] Hayes S. C., Strosahl K. D., Wilson K. G. (2011). Acceptance and commitment therapy: The process and practice of mindful change.

[B30-ejihpe-16-00022] Hayes S. C., Wilson K. G., Gifford E. V., Follette V. M., Strosahl K. (1996). Experiential avoidance and behavioral disorders: A functional dimensional approach to diagnosis and treatment. Journal of Consulting and Clinical Psychology.

[B31-ejihpe-16-00022] Hooper N., Dack C., Karekla M., Niyazi A., McHugh L. (2018). Cognitive defusion versus experiential avoidance in the reduction of smoking behaviour: An experimental and preliminary investigation. Addiction Research and Theory.

[B32-ejihpe-16-00022] Jardin C., Zvolensky M. J., Garey L., Otto M. W. (2015). Examination of smoking inflexibility as a mechanism linking anxiety sensitivity and severity of smoking behavior. The American Journal on Addictions.

[B33-ejihpe-16-00022] Karekla M., Panayiotou G. (2011). Coping and experiential avoidance: Unique or overlapping constructs?. Journal of Behavior Therapy and Experimental Psychiatry.

[B34-ejihpe-16-00022] Karekla M., Panayiotou G., Collins B. N. (2017). Predictors of urge to smoke under stressful conditions: An experimental investigation utilizing the PASAT-C task to induce negative affect in smokers. Psychology of Addictive Behaviors.

[B35-ejihpe-16-00022] Kingston J., Clarke S., Remington B. (2010). Experiential avoidance and problem behavior: A mediational analysis. Behavior Modification.

[B36-ejihpe-16-00022] Klanecky A., McChargue D. E., Bruggeman L. (2012). Desire to dissociate: Implications for problematic drinking in college students with childhood or adolescent sexual abuse exposure. American Journal on Addictions.

[B37-ejihpe-16-00022] Koob G. F., Volkow N. D. (2016). Neurobiology of addiction: A neurocircuitry analysis. The Lancet Psychiatry.

[B38-ejihpe-16-00022] Lappalainen P., Granlund A., Siltanen S., Ahonen S., Vitikainen M., Tolvanen A., Lappalainen R. (2014). ACT Internet-based vs. face-to-face? A randomized controlled trial of two ways to deliver acceptance and commitment therapy for depressive symptoms: An 18-month follow-up. Behaviour Research and Therapy.

[B39-ejihpe-16-00022] Lee E. B., An W., Levin M. E., Twohig M. P. (2015). An initial meta-analysis of acceptance and commitment therapy for treating substance use disorders. Drug and Alcohol Dependence.

[B40-ejihpe-16-00022] Lee E. S., Bong E. J. (2018). Impact of Parents’ problem drinking on suicidal ideation of their university student children: The multiple mediating effects of childhood trauma, experiential avoidance and depression. Journal of Korean Academy of Nursing.

[B41-ejihpe-16-00022] Levin M. E., Lillis J., Seeley J., Hayes S. C., Pistorello J., Biglan A. (2012). Exploring the relationship between experiential avoidance, alcohol use disorders, and alcohol-related problems among first-year college students. Journal of American College Health.

[B42-ejihpe-16-00022] Linehan M. M. (2015). DBT^®^ skills training manual.

[B43-ejihpe-16-00022] Luoma J. B., Pierce B., Levin M. E. (2020). Experiential avoidance and negative affect as predictors of daily drinking. Psychology of Addictive Behaviors.

[B44-ejihpe-16-00022] Mak Y. W., Loke A. Y., Leung D. Y. P. (2021). Acceptance and commitment therapy versus social support for smoking cessation for people with schizophrenia: A randomised controlled trial. Journal of Clinical Medicine.

[B45-ejihpe-16-00022] Martínez-Vispo C., López-Durán A., Senra C., Rodríguez-Cano R., Del Río E. F., Becoña E. (2020). Environmental reward and depressive symptoms in the relationship between avoidance and cigarette dependence in treatment-seeking smokers. Psicothema.

[B46-ejihpe-16-00022] Naifeh J. A., Tull M. T., Gratz K. L. (2012). Anxiety sensitivity, emotional avoidance, and PTSD symptom severity among crack/cocaine dependent patients in residential treatment. Cognitive Therapy and Research.

[B47-ejihpe-16-00022] Page M. J., McKenzie J. E., Bossuyt P. M., Boutron I., Hoffmann T. C., Mulrow C. D., Shamseer L., Tetzlaff J. M., Akl E. A., Brennan S. E., Chou R., Glanville J., Grimshaw J. M., Hróbjartsson A., Lalu M. M., Li T., Loder E. W., Mayo-Wilson E., McDonald S., Moher D. (2021). The PRISMA 2020 statement: An updated guideline for reporting systematic reviews. BMJ.

[B48-ejihpe-16-00022] Paulus M. P., Stewart J. L. (2014). Interoception and drug addiction. Neuropharmacology.

[B49-ejihpe-16-00022] Robles Z., Garey L., Hogan J., Bakhshaie J., Schmidt N. B., Zvolensky M. J. (2016). Examining an underlying mechanism between perceived stress and smoking cessation-related outcomes. Addictive Behaviors.

[B50-ejihpe-16-00022] Serowik K. L., Orsillo S. M. (2019). The relationship between substance use, experiential avoidance, and personally meaningful experiences. Substance Use and Misuse.

[B51-ejihpe-16-00022] Sheynin J., Myers C. E., Ghafar F., Morris A. N., Morley K. C., Haber P. S., Moustafa A. A. (2019). A pilot study of escape, avoidance, and approach behaviors in treated alcohol-dependent males. Journal of Clinical and Experimental Neuropsychology.

[B52-ejihpe-16-00022] Shorey R. C., Gawrysiak M. J., Elmquist J., Brem M., Anderson S., Stuart G. L. (2017). Experiential avoidance, distress tolerance, and substance use cravings among adults in residential treatment for substance use disorders. Journal of Addictive Diseases.

[B53-ejihpe-16-00022] Stotts A. L., Vujanovic A., Heads A., Suchting R., Green C. E., Schmitz J. M. (2015). The role of avoidance and inflexibility in characterizing response to contingency management for cocaine use disorders: A secondary profile analysis. Psychology of Addictive Behaviors.

[B54-ejihpe-16-00022] Twohig M. P., Shoenberger D., Hayes S. C. (2007). A preliminary investigation of acceptance and commitment therapy as a treatment for marijuana dependence in adults. Journal of Applied Behavior Analysis.

[B55-ejihpe-16-00022] Ulusoy S., Ramakan E. D., Gulec V., Alniak I., Yavuz K. F. (2022). Do psychological inflexibility and self-criticism mediate the relationship between depression and addiction severity?. Dusunen Adam Journal of Psychiatry and Neurological Sciences.

[B56-ejihpe-16-00022] United Nations Office on Drugs and Crime (UNODC) (2022). El Informe Mundial sobre las Drogas 2022 de la UNODC destaca las tendencias del cannabis posteriores a su legalización, el impacto ambiental de las drogas ilícitas y el consumo de drogas entre las mujeres y las personas jóvenes.

[B57-ejihpe-16-00022] Vernig P. M., Orsillo S. M. (2009). Psychophysiological and self-reported emotional responding in alcohol-dependent college students: The impact of brief acceptance/mindfulness instruction. Cognitive Behaviour Therapy.

[B58-ejihpe-16-00022] Watson N. L., Heffner J. L., McClure J. B., Bricker J. B. (2017). Relationships between social anxiety and smoking-specific experiential avoidance. Journal of Dual Diagnosis.

[B59-ejihpe-16-00022] World Health Organization (2023). Cannabis.

[B60-ejihpe-16-00022] World Health Organization (2024). Global status report on alcohol and health and treatment of substance use disorders.

